# The epidemiology of wrist and hand injury in two hospitals in Jerusalem: substantial differences between population subgroups

**DOI:** 10.1186/s13584-018-0278-0

**Published:** 2019-01-09

**Authors:** Shai Luria, Daniel Talmud, Ido Volk, Meir Liebergall, Ronit Calderon-Margalit

**Affiliations:** 1 Department of Orthopedic Surgery, Hadassah-Hebrew University Medical Center, Kiryat Hadassah, POB 12000, 91120 Jerusalem, Israel; 2 Braun School of Public Health and Community Medicine, Hebrew University, Jerusalem, Israel

**Keywords:** Hand trauma, Trauma epidemiology, Injury risk factors, Primary prevention, Questionnaires, Emergency department, Cultural and linguistic diversity

## Abstract

**Background:**

Wrist and hand injuries are common and constitute a major economic burden. General injury prevention programs have failed to demonstrate a decrease in injury rates. We hypothesized that there are differences in injury patterns in culturally diverse subpopulations of a metropolitan area treated within the same medical system, which may partly explain the difficulties associated with injury prevention.

**Methods:**

We conducted a survey of patients admitted to emergency departments of two hospitals in Jerusalem for wrist and hand injuries during a 3 month period. Patients were asked to complete a questionnaire regarding demographic data, injury type and mechanism. Injury type and mechanism were then compared for age, gender, level of education and degree of religiosity.

**Results:**

The questionnaire was completed by 799 patients (response rate 62%; 75% male; average age 27). Thirty-one percent reported they were injured at work, 33% at home and 36% during leisure activities. Data analysis showed that several subpopulations were found to be at risk as compared to their corresponding groups and relative proportion in the overall population of the city. These included contusions after falls in non-ultra-Orthodox Jewish women aged 65 years and over, crush injuries in ultra-Orthodox Jews under the age of 10 (53% vs. 14% for non ultra-Orthodox Jews, respectively) and Muslim teens. Muslims were injured more, especially at work, in comparison to their relative proportion in the population as a whole.

**Conclusion:**

Different subpopulations at risk and different injury patterns of wrist and hand injuries were found in this culturally heterogeneous population. Awareness of these differences may be the first step when designing specific injury prevention programs in a culturally diverse population. A combined effort of community leaders and government agencies is needed to deal with the specific populations at risk, although legislation may be needed to limit some of the risks such as teens and specific work related hazards and exposures.

## Background

Wrist and hand injuries are commonly associated with pain, chronic disability, loss of productivity and decrease in quality of life [1–3]. The type and mechanism of injury affect the treatment plan as well as patients’ prognosis considerably. In the Netherlands, 42% of all emergency department visits were due to upper extremity injuries, of which injuries to the wrist and hand were the most common [4]. It has been estimated that wrist and hand injuries rank the highest in cost, since they are both common and carry the indirect cost of productivity loss [2].

Few studies have been published on the epidemiology, determinants and distribution of hand and wrist injuries. These have indicated considerable geographic differences in the settings in which hand injuries take place [1, 5, 6]. For example, workplace injuries were the most common hand injuries in both Turkey [1] and Singapore [6]; however, in Denmark, leisure time injuries were the most common [5].

There has been extensive examination of hospitalized trauma and epidemiological factors on a national scale, using the Israeli National Trauma Registry [7, 8]. The large majority of hand and wrist trauma patients are released form the emergency rooms for further community care and are not hospitalized. These patients are therefore not represented in this registry.

The trauma registry has demonstrated nationwide differences between ethnic subpopulations in Israel, in different trauma types [9, 10]. Ethnicity is a well-known factor in the work force in Israel, including differences in rate of manual labor, women participation in the workforce, job security and other factors [11–13]. Our hypothesis was that in hand trauma patients, there are significant differences between subpopulations within the same municipality, not only in comparison with other countries or on a national scale. Finding these differences in wrist and hand injuries between subpopulations and settings within a specific geographic area, may be crucial to the development of effective prevention programs [14, 15]. We therefore aimed to characterize hand and wrist injuries in Jerusalem concerning subpopulations within the city.

## Methods

We conducted a survey of all patients who came to the emergency departments (ED) of two hospitals in Jerusalem - a large trauma center and a smaller hospital - during a 3 month period between April and June 2013. These two hospitals are two of the three centers in this city that treat orthopedic trauma, are affiliated to each other and to the university medical school. Health care in the city (as in the entire country) is universal and affiliation to a medical insurance plan is compulsory. Each of the hospital’s catchment area includes the entire city of Jerusalem and not limited by city districts. The hospitals are easily accessible by different means of transportation. All trauma admissions to emergency departments in Israel are free according to the Health Ministry criteria, for all citizens.

Inclusion criteria for this study were all patients with acute trauma of any kind, from the mid-forearm to the hand, who agreed to participate. The data was collected specifically for this study, using a questionnaire developed and pilot tested for this purpose. The questionnaire was completed by the patients or accompanying persons (in Hebrew, Arabic or English). In order to collect as much data as possible, the list of all trauma patients seen in the emergency departments of both hospitals (*n* = 4242) was scanned for patients treated for wrist and hand injuries (*n* = 1294) to identify those who did not fill out the questionnaire; these patients were contacted and interviewed on the phone within 2 weeks of the injury by 2 research assistants fluent in the 3 languages. All patients had contact information. Two attempts were made to contact each patient at different dates. Of the 1294 patients, 808 patients agreed to participate (712 questionnaires completed in the emergency departments and 96 completed by phone interviews; total of 62% response rate). The 38% who did not participate, were not asked to participate by the physician on call, did not wish to participate, or could not be contacted by phone later. Of these patients, 78% were male and 55% were Jews which is comparable to the cohort of patients interviewed. Information on their degree of religiosity or the type/mechanism of the injury could not be accurately assessed. During the course of the study, the main factor decreasing the response rate was the cooperation of the physician on call and not patients factors. This was usually due to physician lack of motivation or periods of increased workloads during a shift. Therefore, we believe the cohort of patients was representative of the entire patient group.

The final questionnaire was prepared on the basis of the results of a pilot questionnaire. Participants indicated information on their background as well as details about the injury. This included gender, age, years of education, religion and degree of religiosity. Degree of religiosity was included because it constitutes a major factor of social identification in the Jewish community. All patients were asked to indicate their profession, number of years in their current job and the setting in which the injury occurred: work/school/military service, home, leisure (outside the home). The patients selected the type of the injury from a detailed list (Table 2).

The official demographic data for the city of Jerusalem and its working force is available online [11–13, 16, 17]. As reference to our results, we presented the official demographic data which most complied with the geographic area and period of the study, although the accuracy of congruence was not precise or could not be verified at times.

The Hadassah institutional ethics committee approved the study protocol.

### Study variables

The questionnaire collected detailed information on mechanism of injury (for example: door slamming, ball injuries, falls, etc.). We further grouped these detailed descriptions into five major types of mechanisms of injury (contusion, laceration, crush, explosion or burn) based on the epidemiological literature [1, 3, 6, 18]. This classification was designed to group the patients into clinically meaningful types, which would have implications for injury prevention programs. We obtained limited data on the type of occupation and could only characterize the professions roughly into 1) office or domestic or 2) construction, industry or farming.

Patients’ age was subdivided into three groups (under 17, 17–65 and over 65) and further into 6 age brackets (under 10 years, 11–16, 17–24, 25–40, 41–65 and over 65). The first classification broke down the sample into children and teens, the labor force and older patients. The more detailed classification aimed to make a distinction between younger children, teens and young adults. Level of education was divided into 3 groups (0–8 years, 9–12 years or 13 years or more, corresponding to elementary school, high school, and higher education, respectively).

Degree of religiosity was rated in the Jewish patients according to the patients’ self-reported level of religious observance into ultra-Orthodox, traditional, conservative or secular [12].

### Data analysis

Distributions of the type and mechanism of injury were constructed in terms of age, gender, levels of education, degree of religiosity and setting of the injury. These groups were compared using Pearson Chi-square. Two-tailed *p* values of < 0.05 were considered statistically significant. All analyses were carried out using the SPSS statistical package, version 21.

## Results

Of the 808 participants, injury type was documented in 799, which thus constituted the total number of questionnaires for analyses. Of these patients, 74.5% were males, a much higher proportion than in the city population (Table 1). Female patients were older than male patients (mean age at admission: 34 *±* 25 vs. 25 *±* 16 years, respectively). Jews were the majority and constituted 54.7% of the patients (437 patients). Muslims made up 41.4% of the patients (331), a higher proportion than in the city population. The mechanism of injury was documented in 775 patients (97%). We did not find differences between phone and ED interviews between religion or religiosity groups. Women were slightly more likely to be interviewed on the phone (*p* = 0.047).

**Table 1:** Characteristics of the study population according to injury type

		Contusion	Laceration	Crush	Explosion	Burn	Total – n (% of total)	Statistical significance (between groups)*	Representation in city population (%)
Total - n (% of total)		448 (56.1%)	211 (26.4%)	115 (14.4%)	7 (.9%)	18 (2.2%)	799 (100%)		
Sex – n (% of sex)	Male	324 (54.4%)	161 (27.1%)	92 (15.4%)	5 (.8%)	13 (2.2%)	595 (74.5%)	ns.	49% [10]
	Female	124 (60.8%)	50 (24.5%)	23 (11.3%)	2 (1%)	5 (2.5%)	204 (25.5%)		51% [10]
Age group – n (% of age group)	16 or younger	158 (58.3%)	48 (17.7%)	**59 (21.8%)**	1 (.4%)	5 (1.8%)	271 (33.9%)	*p* < 0.0001	37% [9]
	17 to 65 years	258 (53.3%)	**157 (32.4%)**	50 (10.3%)	**6 (1.2%)**	**13 (2.7%)**	484 (60.6%)		54% [9]
	Over 65 years	32 (72.7%)	6 (13.6%)	6 (13.6)	0 (0)	0 (0)	44 (5.5%)		9% [9]
Level of education (for patients over age 18) – n (% of education level)	0–8 years	41 (45.6%)	38 (42.2%)	8 (8.9%)	1 (1.1%)	2 (2.2%)	90 (22.6%)	ns.	66% [11]
	9–12 years	96 (54.5%)	55 (31.3%)	16 (9.1%)	3 (1.7%)	6 (3.4%)	176 (44.2%)		
	13 years or more	75 (56.8%)	39 (29.5%)	14 (10.6%)	1 (.8%)	3 (2.3%)	132 (33.2%)		34% [11]
Religion – n (% of religion)	Jewish	254 (58.1%)	99 (22.7%)	71 (16.2%)	4 (.9%)	9 (2.1%)	437 (54.7%)	ns.	64% [9]
	Muslim	174 (52.6%)	102 (30.8%)	43 (13.0%)	3 (.9%)	9 (2.7%)	331 (41.4%)		34% [9]
Jewish Religiosity – n (% of Jewish religion)	Ultra-Orthodox	**30 (39.0%)**	**22 (28.6%)**	**24 (31.2%)**	**0 (0%)**	1 (1.3%)	77 (17.6%)	*p* = 0.008	35% [9]
	Religious	58 (60.4%)	22 (22.9%)	12 (12.5%)	1 (1%)	3 (3.1%)	96 (22%)		31% [9]
	Traditional	79 (60.8%)	29 (22.3%)	20 (15.4%)	2 (1.5%)	**0 (0)**	130 (29.7%)		
	Secular	85 (65.4%)	25 (19.2%)	15 (11.5%)	1 (.8%)	4 (3.1%)	130 (29.7%)		31% [9]
Setting – n (% of setting)	Work/army/ school	120 (49.8%)	75 (31.1%)	37 (15.4%)	**4 (1.7%)**	5 (2.1%)	241 (30.1%)	*p* < 0.001	
	Home	116 (45.7%)	**86 (33.9%)**	**47 (18.5%)**	1 (.4%)	4 (1.6%)	254 (31.2%)		
	Leisure	**202 (72.1%)**	44 (15.7%)	24 (8.6%)	2 (.7%)	**8 (2.9%)**	280 (35%)		

Religion was other than Jewish or Muslim in 17 patients and not reported in 14 of the patients. Religiosity was not reported for four Jewish patients. Setting was not reported in 24 patients

*Ns*. Not significant

*Pearson Chi-Square

Data marked in bold emphasis to pinpoint the differences between the data sets

### Gender

**Fig. 1:**
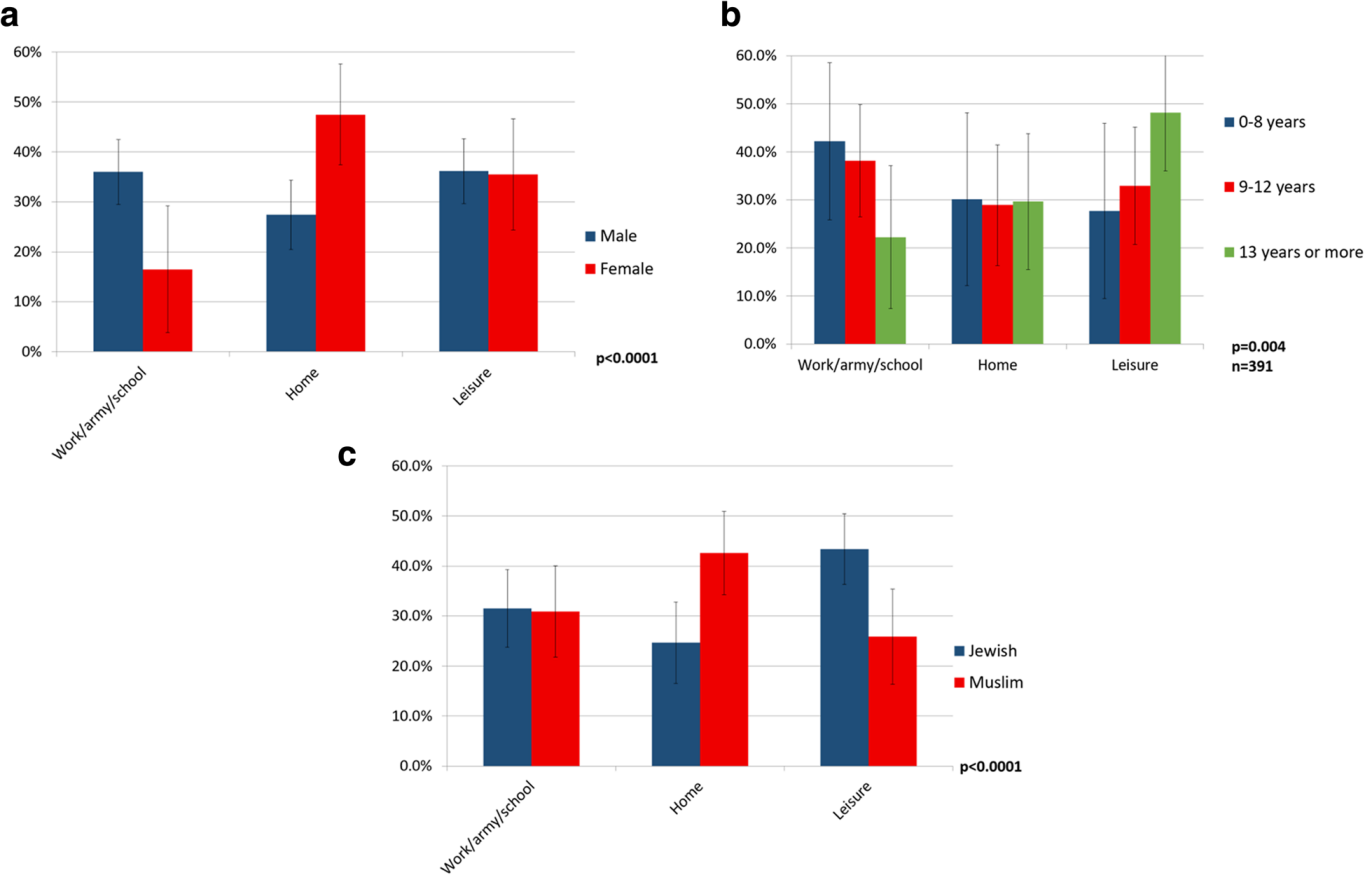
The injury setting was found to differ significantly between genders (**a**) and religious groups (**b**). In patients over the age of 18, the setting was also found to differ according to the level of education (**c**). The significant results show the dependence between the variables. Results presented with *95*% confidence interval error bars

The results showed significant associations between gender and age group as well as between gender and setting (Fig. 1a). Forty-eight percent of females were injured at home (compared with 27% of males) and 36% of males were injured at work (compared with 17% of females) (*p* < 0.0001). Thirty-six percent of males and 35% of females were injured during leisure activities out of the house. Female patients were more commonly injured under the age of 10 or over 40. Fifteen percent of the women examined were over the age of 65 as compared to 2% of the men (*p* < 0.001) (Jerusalem city statistics indicate 11% of all females and 9% of all males were over 65 in 2013 [13]). Falls were the most common cause of injury in both genders although it was much more common in females (30.6% vs. 49.5% respectively; *p* < 0.0001 for gender differences across all injury mechanisms) (Table 2).

**Table 2:** Characteristics of the study population by mechanism of injury

Mechanism of Injury		Fall	Door slamming	MVA	Knife cut	Ball injury	Hit by falling object	Bruised by other person	Burn	Saw/ hammer injury	Bite	Bullet injury	Other	Total - n (% of total)	Statistical significance
n (% of total)		274 (33.9%)	81 (10%)	42 (5.2%)	50 (6.2%)	30 (3.7%)	47 (5.9%)	20 (2.5%)	17 (2.1%)	28 (3.4%)	10 (1.2%)	4 (0.5%)	172 (21.3%)	775 (97%)	
Sex	Male	177 (30.6%)	61 (10.5%)	**34 (5.9%)**	38 (6.6%)	**27 (4.7)**	**43 (7.4)**	**17 (2.9)**	12 (2.1%)	**28 (4.8%)**	8 (1.4%)	**4 (.7%)**	130 (22.5%)	579 (72.5%)	X^2^ value - 38.122 df - 12 *p* < 0.0001*
	Female	**97 (49.5%)**	20 (10.2%)	8 (4.1%)	12 (6.1%)	3 (1.5%)	4 (2%)	3 (1.5%)	5 (2.6%)	0 (0)	2 (1%)	0 (0)	42 (21.4%)	196 (24.5%)	
Age	16 or younger	109 (41.3%)	**48 (18.2%)**	2 (.8%)	8 (3%)	**18 (6.8%)**	10 (3.8%)	3 (1.1%)	5 (1.9%)	2 (.8%)	**6 (2.3%)**	0 (0)	53 (20.1%)	264 (33%)	X^2^ value - 112.898 df - 24 *p* < 0.0001*
	17 to 65 years	137 (29.3%)	28 (6%)	**39 (8.3%)**	**41 (8.8%)**	12 (2.6%)	**37 (7.9%)**	**16 (3.4%)**	**12 (2.6%)**	**25 (5.3%)**	4 (.9%)	**4 (.9%)**	**113 (24.1%)**	468 (58.6%)	
	Over 65 years	**28 (65.1%)**	5 (11.6%)	1 (2.3%)	1 (2.3%)	0 (0)	0 (0)	1 (2.3%)	0 (0)	1 (2.3%)	0 (0)	0 (0)	6 (14.0%)	43 (5.4%)	
Religion	Jewish	147 (34.1%)	46 (10.7%)	**30 (7%)**	28 (6.5%)	**23 (5.3%)**	21 (4.9%)	**14 (3.2%)**	9 (2.1%)	7 (1.6%)	**6 (1.4)**	1 (0.2%)	99 (23%)	431 (53.9%)	X^2^ value – 32.081 df – 12 *p* < 0.001*
	Muslim	119 (37.7%)	34 (10.8%)	11 (3.5%)	19 (6%)	4 (1.3%)	**26 (8.2%)**	5 (1.6%)	8 (2.5%)	**20 (6.3%)**	3 (0.9)	3 (0.9%)	64 (20.3%)	316 (39.5%)	

In 24 patients, although the type of injury was clear, the exact mechanism was not recorded

*MVA* Motor vehicle accident

*Pearson Chi-Square

Data marked in bold emphasis to pinpoint the differences between the data sets

### Age and level of education

Distribution of type of injury differed by age group. Contusion was the most common in all age groups, especially among the elderly (58, 53, and 73% in the 0–16, 17–65, and > 65 groups, respectively; *p* < 0.001 for type of injury) (Table 1). Patients over the age of 65 were more likely to be injured by falls and children under the age of 17 were more likely to suffer from crush injuries by a door, a ball, or a bite (Table 2).

For patients over the age of 18 (*n* = 398), there was a significant association between the level of education and the setting of the injury (*p* = 0.004 for level of education) (Fig. 1b). By contrast, there was no association between level of education and injury type or mechanism.

### Religion

Eighteen percent of the Muslim participants were between the ages of 11 and 16 (vs. 11% Jewish participants) and 9% of the Jewish participants were over the age of 65 (vs. 2% of the Muslims) (*p* = 0.003 for religion). By comparison, in 2013, the population of Jerusalem in the 11–16 age bracket was, 14% Muslims and 11% Jews. The 65 and over bracket was composed of 4% Muslims and 11% Jews [12].

The Jewish patients reported more injuries during leisure activities (43%) whereas Muslims experienced more injuries at home (43%) (*p* < 0.0001 for injury setting) (Fig. 1c). The percentage of work injuries was similar (31%) (although the work force in Jerusalem in 2012 was composed of 40% of Muslims and 56% of Jews. The male workforce in 2012 was 67% of Muslim men and 52% of Jewish men [12].

Of the patients 18 or older (*n* = 398), 78% of the Jews and 84% of the Muslims reported their occupation. Of these, 26% of the Muslims and 17% of the Jews reported working in industry or construction. Similar proportions of Muslims and Jews reported having office or domestic jobs (58 and 61%, respectively). This was similar to national figures [11] although there are gender differences that are beyond the scope of this study. Jewish patients reported significantly longer tenure in the workplace than the Muslim patients did (10.5 years for Jews vs. 6.3 years for Muslims; *p* = 0.028). Significant differences were found between Jews and Muslims when examining specific injury mechanisms (Table 2) (*p* = 0.001 for distribution of injury mechanism).

### Degree of religiosity

**Fig. 2:**
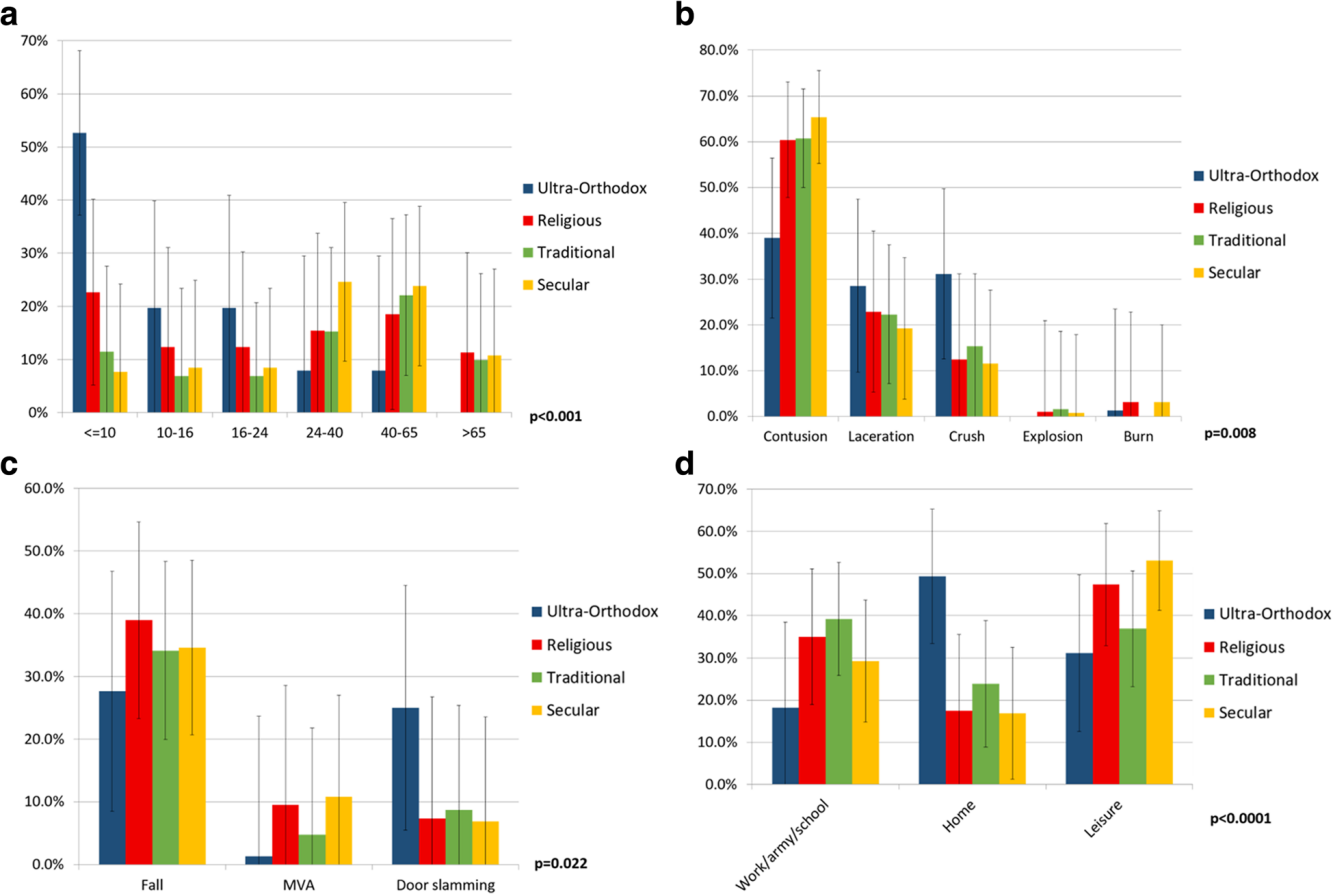
Significant differences between different segments of the Jewish population according to reported degree of religiosity for age groups (**a**), injury type (excluding explosion and burn injuries) (**b**) and mechanism of injury (**c**, most frequent mechanism presented) and the injury setting (**d**). Explosion and burn injuries were uncommon in this population. Burn injuries were occurred in 1, 2 and 1 patients in the Religious, Traditional and Secular patients, respectively. Explosion injuries occurred in 1, 3 and 4 patients in the ultra-Orthodox, Religious and Secular patients, respectively. The significant results show dependence between the variables. Results presented with *95*% confidence interval error bars

Among Jews, more than 50% of the ultra-Orthodox patients were under the age of 10 (*p* = 0.022 for religiosity groups) (Fig. 2a). This rate differed from the city’s population, where 37% of the ultra-Orthodox and 17% of the remainder of the Jewish population were under the age of 10 in 2013 [16]. There were no injuries in ultra-Orthodox Jews over 65 (versus 4% ultra-Orthodox and 12% of other Jews over the age of 65 in the total city population) [16]. Ultra-Orthodox Jews differed from the other Jewish religious groups in terms of injury type (31% crush injuries; *p* = 0.008 for religious groups) (Fig. 2b), mechanism of injury (25% from door slamming; p = 0.022 for religious groups) (Fig. 2c) and setting (49% injured at home; *p* < 0.0001 for religious groups) (Fig. 2d).

## Discussion

Examining hand and wrist acute trauma in two major centers in one metropolitan area during a period of 3 months, we found several groups that were injured more than would be predicted in terms of their relative proportion in the city population. The most significant differences in injury patterns were found between social/religious groups. Over the age of 65, a higher rate of non-ultra-Orthodox Jewish females were injured by falls. It is well known that wrist fractures are common among post-menopausal women [19]. This may not be true in our population of ultra-Orthodox Jewish or Muslim women. It is possible that the women in these population groups fall, but do not suffer from fractures or injuries that call for ED care. It is our experience that with significant injury such as distal radius fractures, female patients from all populations groups will seek medical care in the ED. Other possible explanations for these differences could be cultural, such as activity preferences or rate of participation in higher risk activities, but could also be related to differences in the rate of osteoporosis in genetically diverse populations or even dietary preferences in different communities [20]. There is no specific information regarding these differences in the literature in this community. Differences have been reported between ethnicity groups in risk of osteoporosis or low bone mineral density levels measured in the hip or spine [21–23]. Reports demonstrated differences between Jews and Arabs in Israel [24] and Ultra-orthodox teens in Brooklyn [25]. Multiple causes have been suggested to explain this, including different DEXA machines. No conclusive data regarding these factors has been demonstrated [23–25].

A higher proportion of Muslims teens were injured between the ages of 11–16, compared to the Jewish population. Previous reports revealed a high rate of burn injury in this age group, on a national scale [10, 26]. The authors speculate that there are differences in the family role of the teenager in Muslim and Jewish families. In the Muslim family, the teen is regarded as an adult, therefore at risk of injury as a young adult, including at work [26]. We found that more Muslim males were injured at work relative to their proportion in the working population [11]. This may be explained by a the fact that 55% of Arab males in Jerusalem are manual or unskilled workers according to published reports (in comparison with 17% of the Jewish male population) [12]. In our cohort, 26% of Muslims reported manual labor in comparison with 17% of the Jews, although only 84% of Muslims and 78% of the Jews over the age of 18 reported their occupation. The high rate of Muslim patients being injured can be explained by several other findings (that are consistent with local and national demographic data [11]): Muslim patients, compared to Jewish patients, reported fewer years of education, fewer years in their current jobs and a higher rate of manual occupations which are prone to injury (in industry and construction). Among the Muslim patients, 34% had lower levels of education (vs. 15% in the Jewish patients) and 14% had higher levels of education (vs. 47% in the Jewish patients). We found lower levels of education to be related to injury at work, whereas individuals with higher levels of education were injured more often during leisure time activities (Fig. 1c). The lower socioeconomic status of the Muslim population is an additional risk factor [26].

Within the ultra-Orthodox Jewish population, there was an inverse association between age and hand injury. Although this subpopulation has a high fertility rate, the rate of injury was still exceedingly high among children. There was a significantly high rate of crush injuries at home, specifically by door slamming, compared to the other populations. Previously, a high rate of burn injuries has been reported in ultra-Orthodox children [26]. Lack of adult supervision on children in the large families, the lower economic status of this population as well as the common use of exposed heating equipment during the Sabbath, have been reported as possible explanations for the high rates of injury [26].

Differences between religious groups may have little to do with religion per se, but rather with the fact that they represent social groups or even separate communities. In Jerusalem, they are separated into different neighborhoods, go to different community centers, have different community leaders, political parties and newspapers. This may be true in many multi-cultural metropolitan areas, in addition to other factors such as level of income, geographic factors or available employment [1] which we did not evaluate in this study. Regardless of these explanations, we believe that the differences between religiosity groups must be taken into consideration when planning injury prevention programs.

Previous studies of trauma in Israel include extensive analysis of trauma on a national level, based on data from the National Trauma registry [7, 8, 27] or specific medical centers [28]. However, these studies accounted only for hospitalized patients and excluded those who were not hospitalized, which are the majority of patients with hand trauma. In addition, the national registry reports do not include accounts of specific injury groups such as upper extremity or wrist and hand trauma. The representation of large minority populations in the registry has been questioned, specifically populations residing at a greater distance from the large medical centers where the data is collected and getting care at more rural medical centers [29]. The aim of this study was to collect data, which is not represented in the registry or other reports, examining hand trauma specifically. The additional strength of this report is the depiction of cultural diversity of trauma in a multi-cultural, multi-religion metropolitan area. Since accessibility to healthcare is universal on a national scale, our findings suggest that differences between subpopulations may stem from cultural differences related to the role and activities of women, children and the elderly in the community, involvement in leisure activities outside of the home, level of education, and exposure to occupational hazards.

The epidemiology of hand trauma in our population differed in many ways in comparison with other studies in the literature. In a highly industrial area in Turkey [1], work was found to be associated with the highest risk (85% of the patients), whereas only a third of our population was injured at work. Women were less frequently injured then men (17%) in the Turkish study than in ours (25.5%). In the Turkish study contact with machinery was the cause of injury in 47% of the cases with an amputation rate of 32% compared to only 4% leisure time injuries (vs. 35% in our study) [1]. It is possible that there are fewer injuries outside the workplace in the population of this specific area of Turkey although it is also possible that patients with more minor injuries are referred to other clinics or hospitals. In general, we found more similarities between our cohort and that of a Danish report [5] in terms of setting, and type of injury. In the Danish report, 32% were injured at home and 33% at work, in comparison with 31 and 33% in our study, respectively. In the Danish study 23% of patient fell and 5% were injured in traffic accidents, in comparison with 33 and 5% in our study, respectively. The comparison of data collected in different countries and continents is limited by numerous factors, including the medical system structure, thresholds of referral to emergency departments by physicians, as well as by the patients themselves [30] and availability of other medical centers not included in the study [1]. From these differences, it is obvious that if injury prevention programs are planned, the experience in other countries must be taken with reservations. Local programs, tailored to the specific characteristics of our population and aimed at specific subpopulations at risk, are needed.

### Limitations

There are several limitations to this study. It is well-known that there are seasonal changes in trauma patterns [1, 31] although there is no data regarding variations in rate of hand trauma. The data for this study was collected during three consecutive spring months and seasonal changes were not examined.

The questionnaire was simple, pilot tested and available for participants in different languages in order to lower possible errors related to education and language. As with any questionnaire, the answers were the subjective report of the participant, which may pose potential bias, including misrepresentation of information for financial or legal reasons, presumed by the participant. We could not control for this possibility and did not re-examine the reported information.

Thirty-eight percent of the patients either did not fill out the questionnaire in the ED or could not be contacted on the phone. Although this poses a potential selection bias, we do not have reason to believe that less compliance of patients in the ED was related to a specific study group. We found that the differences in compliance was related mainly to the compliance of the treating physician in the ED. It is possible that increased workload during shifts effected physician compliance although this is highly dependent on the physician on call and documentation of changes in workload during a shift was not available. Overall, similar rates of males and Jews were found between participants and the non-responders. There may have been differences in education level, setting of injury or religiosity in non-responders, which were not available to us for comparison. Potentially, this may have affected the proportion of injured individuals in each of the groups and skewed the results, although we believe this to have only a minor impact.

Finally, the data presented here includes only patients referred to the hospital for care, not community care centers. There is no information comparing the epidemiology or injury severity between patients treated in the hospital or in the community. In principle, the hospital treated population represents the more severely injured patients needing specialized urgent care, although the variability between referral practices may be great. There is no available data regarding the referral preferences of community doctors, other than more severe injuries, which are usually directed to the large trauma center that was included in this study. There is also no published data related to patient referral preferences according to religion or cultural groups in Jerusalem.

Examination of the severity of the injury was not an aim of this study, although this may be of importance when deciding what prevention programs should take priority. There are reports in Israel of differences in the utilization of health care between Muslim and Jewish patients, specifically lack of Arab speaking specialists [32]. We do not know if this is true in Jerusalem, where the language barriers at the HMO clinics and hospitals is a minor problem, due to the multilingual staff throughout the city’s healthcare system. In this study, the comparison was made within each religious and religiosity group separately, making the question of health care utilization trends of less importance. We also did not examine specialty clinics but ED patients.

In work related injuries, acute and chronic, customized intervention plans have been shown to be successful in decreasing rates of illness [33, 34]. Improvement in equipment, such as better designed hand knotting carpet looms, may be effective in lowering injury rates [33]. Similar success has been demonstrated with specific leisure time injuries, such as a decrease of finger injuries in contact flag football games, when players are not allowed to wear pants with pockets [15]. It is possible that in our subpopulation of ultra-Orthodox children, simple technical solutions aimed at the prevention of door slamming, may be effective in lowering injury rates. Simple interventions are available for this purpose [35]. Education authority initiatives combined with community leadership are needed to implement such interventions, and these may prove effective in homes as well as in educational institutions. Regulation mandating technical solutions in educational facilities and financial support to enable this may be more efficient in schools.

Introduction of better designed equipment, such as safety devices to prevent needle stick injury in medical staff, have been shown to be more effective when combined with an interactive injury prevention workshop [34]. Company-oriented interventions such as a targeted safety campaigns have been demonstrated to reduce non-lethal injury in construction workers [14]. Educational interventions such as counseling caregivers about better supervision and safe home environment, special rallies and brochures have been recommended, with limited proof of success [27, 36–39]. For the prevention of injury in Muslim teens in our community, these may all be adequate techniques. Taking into account their family role and socioeconomic background, this is probably a difficult goal to achieve. According to a parliamentary report, only 66% of 17-year-old Arabs attend school (compared with 90% of Jewish teens) [40]. This high rate of school dropout leads to the hiring of these teens in unprofessional, industrial jobs. Keeping Muslim teens in schools is a major goal of both the community and authorities. Implementing safety regulations in smaller workplaces, where these teens work, mainly unofficially, is required as well. Legislation, or strict enforcement of regulations, may be one tool that may aid in lowering injury risk in this population. Legislation has been shown to be effective in injury prevention [41]. Berger et al. demonstrated that states in the US that allowed a wide variety of fireworks for personal use had injury rates more than seven times higher than states that did not allow this [41]. This is a prime example of successful injury prevention.

## Conclusion

In this emergency department-based study of patients with acute trauma of the wrist and hand, differences emerged in terms of patterns of injury across gender, age, level of education, religious groups and degree of religiosity. Future studies should confirm these findings, and more importantly aim at planning effective, targeted interventions to prevent these injuries.
